# Supplementary material for: Online speech synthesis using a chronically implanted brain–computer interface in an individual with ALS
Source: Sci Rep. 2024 Apr 26;14:9617. doi: 10.1038/s41598-024-60277-2 (PMC11053081; doi:10.1038/s41598-024-60277-2)
Supplement: Supplementary file 1 — Supplementary Information. [file 41598_2024_60277_MOESM1_ESM.docx]

# Supplementary Materials


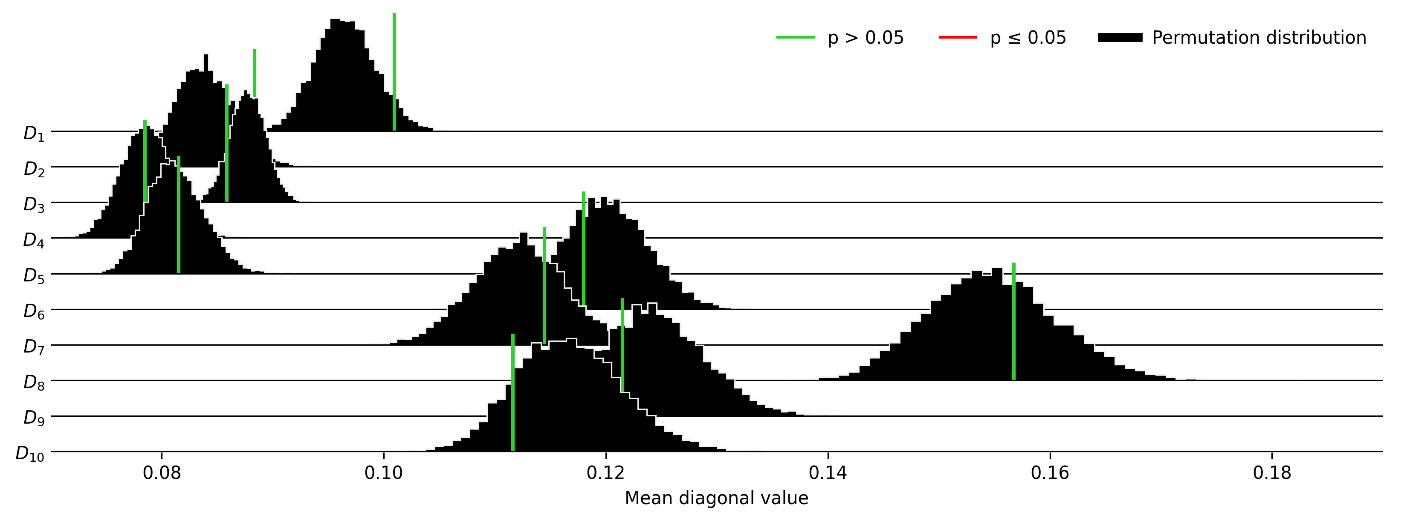
**Supplementary Figure S1 | Acoustic contamination report.** We checked all recordings in our training, validation and test sets for acoustic contamination using the method proposed by Roussel et al.^1^. For each day, the histogram represents mean diagonal values of the distribution of permutated contamination matrices (N=10,000). Colored bars indicate the position of the contamination index of neural recordings, while the statistical criterion for rejecting the null hypothesis is indicated in green (p > 0.05) or in red (p ≤ 0.05) if acoustic contamination is present. After correcting Channel 46 for D_5_, D_6_, and D_7_ we did not detect any acoustic contamination. Recordings of the closed-loop sessions where not checked for acoustic contamination since they were completely withheld from network training procedures, and therefore cannot leak into model training and lead to the Clever Hans phenomenon^2^.


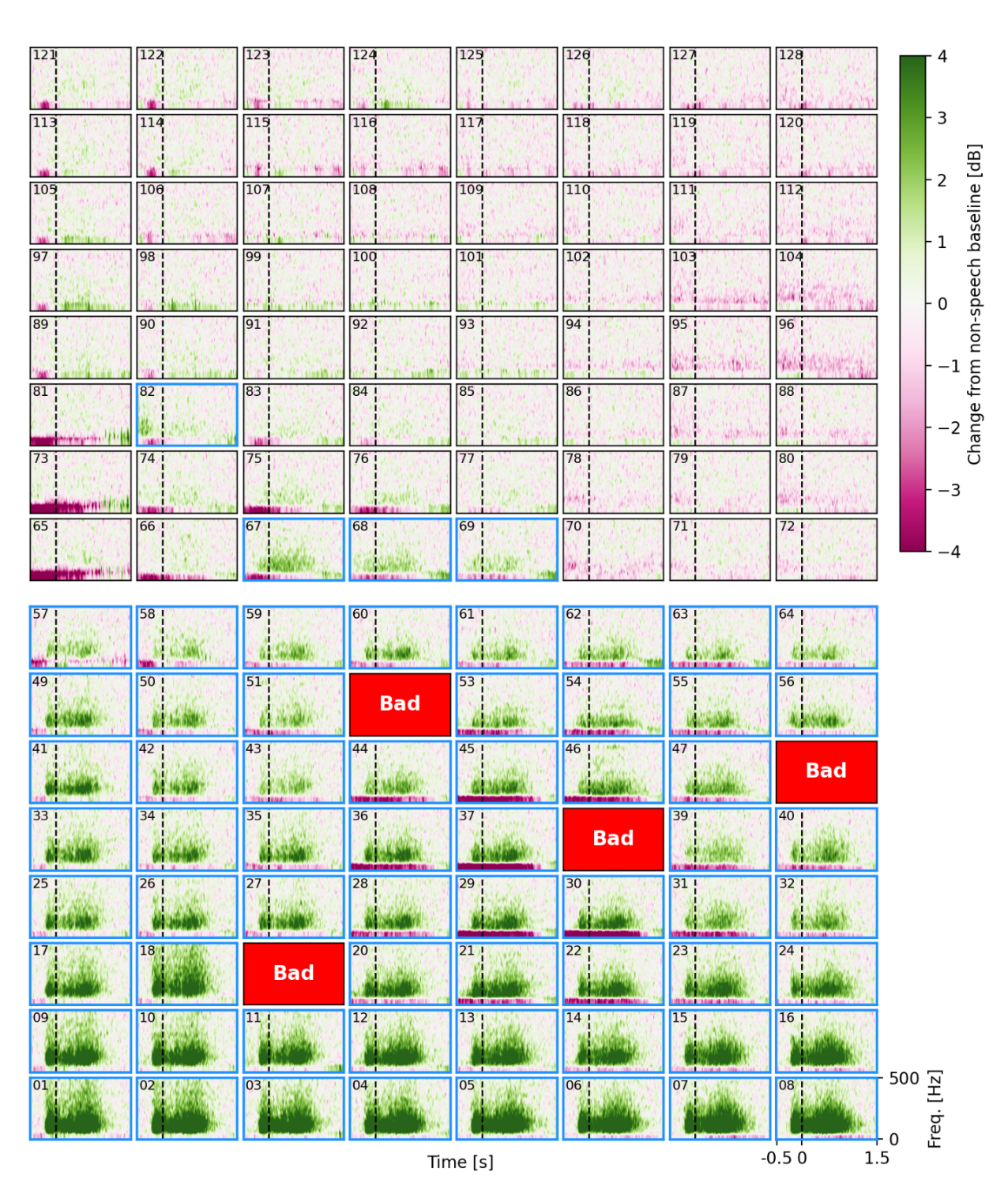
**Supplementary Figure S2 | Power spectral analysis for identifying speech-related channels.** Each plot shows channel specific normalized power spectral density in the frequency range of 0 - 500 Hz averaged across all keywords for a particular recording session in the training set (60 trials). Dashed lines indicate speech onset times, while the x-axis shows time-aligned power spectral density in the range of -0.5 s up to 1.5 s. Bad channels were excluded based on visual inspection. Channels marked with blue borders represent the top-64 channels whose lower bound of a 95% confidence interval most often exceed baseline high-gamma activity (see Supplementary Data for upper and lower bounds, training set only) and were selected as relevant channels for the speech synthesis task.


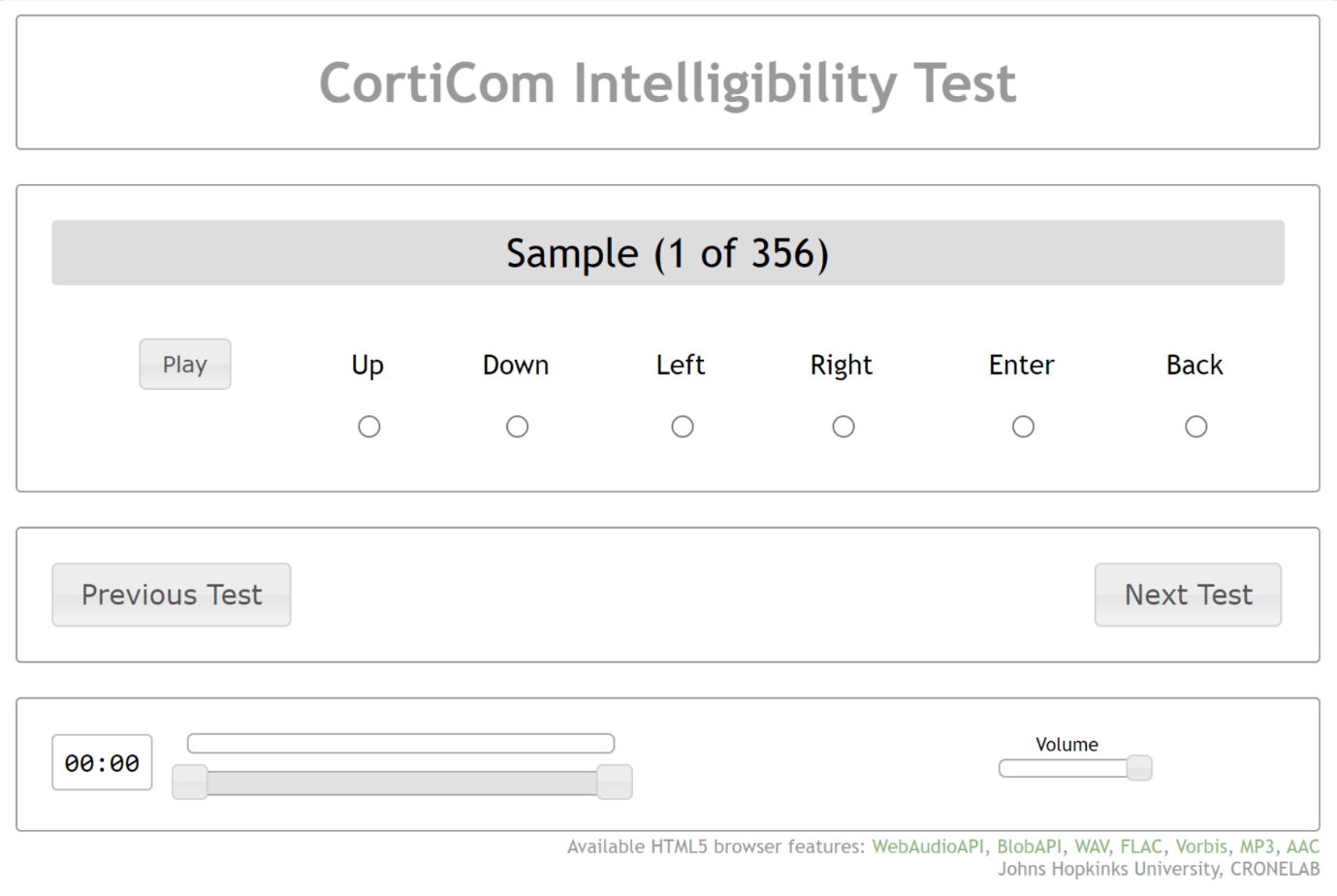
**Supplementary Figure S3 | Web-Interface for conducting the listening test.** All synthesized samples are presented in a randomized sequence. Human listeners can use the Play button to play the sample and subsequently select one of the radio buttons to make their choice or play the sample again. After making a choice they can move on to the next sample. Human listeners can go back to the previous sample if they accidentally make a wrong decision, but not beyond it.

| **Nr.** | 1 | 2 | 3 | 4 | 5 | 6 | 7 | 8 | 9 | 10 | 11 | 12 |
| --- | --- | --- | --- | --- | --- | --- | --- | --- | --- | --- | --- | --- |
| **CV** | ZOO | VOO | HEE | BAA | GEE | THEE | HAA | JEE | LAA | TOO | YAA | MOO |

**Supplementary Table S4 | Syllable stimuli from the syllable repetition task.** Based on Bouchard et al. (Bouchard, 2013) we chose 12 consonant-vowel (CV) syllable stimuli to acquire high-gamma activity of articulatory representations for a stable baseline across days.

**ALS Functional Rating Scale (ALSFRS-R)**

(BOLD number represents level of functioning)

**1. SPEECH**

   4. Normal speech processes

   3. Detectable speech disturbances

   2. Intelligible with repeating

**1. Speech combined w/ nonvocal communication**

   0. Loss of useful speech

**2. SALIVATION**

   4. Normal

   3. Slight but definite excess of saliva in mouth; may have nighttime drooling

   2. Moderately excessive salvia; may have minimal drooling

**1. Marked excess of saliva with some drooling**

   0. Marked drooling; requires constant tissue or handkerchief

**3. SWALLOWING**

   4. Normal eating habits

   3. Early eating problems – occasional choking

   2. Dietary consistency changes

**1. Needs supplemental tube feedings**

   0. NPO (exclusively parenteral or enteral feeding)

**4. HANDWRITING**

   4. Normal

   3. Slow or sloppy; all words are legible

   2. Not all words are legible

**1. Able to grip pen but unable to write**

   0. Unable to grip pen

**5a. CUTTING FOOD AND HANDLING UTENSILS**

   (Patients *without* gastrostomy) **NA**

   4. Normal

   3. Somewhat slow and clumsy; no help needed

   2. Can cut most foods (clumsy and slow); some help needed

   1. Foods must be cut by someone, but can still feed slowly

   0. Needs to be fed

**5b. CUTTING FOOD AND HANDLING UTENSILS**

   (Patients *with* gastrostomy)

   4. Normal

   3. Clumsy, but able to perform all manipulations independently

**2. Some help needed with closures and fasteners**

   1. Provides minimal assistance to caregiver

   0. Unable to perform any aspect of task

**6. DRESSING AND HYGIENE**

   4. Normal

   3. Independent self-care with effort or decreased efficiency

**2. Intermittent assistance or substitute methods**

   1. Needs attendant for self-care

   0. Total dependence

**7. TURNING IN BED & ADJUSTING BEDCLOTHES**

   4. Normal

   3. Somewhat slow and clumsy, but no help needed

**2. Can turn alone or adjust sheets, with great difficulty**

   1. Can initiate, but not turn or adjust sheets alone

   0. Helpless

**8. WALKING**

**4. Normal**

   3. Early ambulation difficulties

   2. Walks with assistance

   1. Nonambulatory functional movement only

   0. No purposeful leg movement

**9. CLIMBING STAIRS**

**4. Normal**

   3. Slow

   2. Mild unsteadiness or fatigue

   1. Needs assistance

   0. Cannot do

**10. DYSPNEA** (shortness of breath)

   4. None

**3. Occurs when walking**

   2. Occurs with one or more of the following: eating, bathing, dressing

   1. Occurs at rest, either sitting or lying

   0. Significant difficulty, considering using mechanical support

**11. ORTHOPNEA** (trouble breathing when lying flat)

   4. None

**3. Some difficulty sleeping at night due to shortness of breath; does not routinely use more than 2 pillows**

   2. Needs extra pillows in order to sleep (more than 2)

   1. Can only sleep sitting up

   0. Unable to sleep

**12. RESPIRATORY INSUFFICIENCY**

   4. None

   3. Intermittent use of BiPAP/NIV

**2. Continuous use of BiPAP/NIV during the night**

   1. Continuous use of BiPAP/NIV during the night and day

   0. Mechanical ventilation (intubation or tracheostomy)

**Total: 26/48**

**Supplementary Data S5 | ALSFRS-R evaluation.** The assessment for the ALSFRS-R measure^3^ has been obtained by the clinical care team at the Johns Hopkins Hospital one day prior to when we began collecting training data. Overall, our clinical trial participant achieved a rating of 26 out of 48 points, where only 1 point contributed from his remaining speech capabilities.

**Supplementary Note | Speech capability assessment of the participant.** A certified speech-language pathologist with extensive experience evaluating and treating individuals with neurogenic communication and swallowing disorders assessed speech/oral motor function using a functional component approach. Oral/speech and nonspeech tasks were employed to assess the functional components of respiration, phonation, resonance, and articulation. Remarkable oral/nonspeech motor findings included weak cough on command; bilateral velar droop at rest and reduced velar elevation during phonation; lingual atrophy, limited lingual range of motion during protrusion, lateralization and elevation, decreased lingual protrusion and lateralization against resistance bilaterally; decreased labial range of movement during retraction and pucker and diminished labial seal against. Mandibular range of movement and strength against resistance were preserved. Speech production was primarily characterized by hypernasality with nasal emission of air. Articulation in connected speech was imprecise with all phonemes being nasalized. Oral/nasal contrasts were not maintained during production of minimal pairs. Spontaneous speech also was low volume phonation although the patient was able to increase vocal loudness on command for limited speech output. Phonatory quality was breathy, somewhat strained, and intermittently wet-hoarse vocal. There were also occasional phonation breaks. Inhalations and exhalations did not occur at appropriate linguistic junctions, resulting in short phrasing in sentences. Objective speech measures were obtained. Maximum sustained phonation was 6.79 seconds for the voiced phoneme /a/; maximum sustained production of the voiceless phoneme /s/ was less than 2 seconds; both values are below the expected duration of at least 10 seconds^4^. Alternating and sequenced movements were reduced in rate (i.e., 6 repetitions of /p˄/ in 5 seconds; 5 repetitions of /t˄/; 7 repetitions of /k˄/ in 5 seconds; 4 repetitions of “Topeka” in 5 seconds)^5^. Rate of speech was slow and labored with reading rate equal to 50 words/minute (expected rate = 150-190 words/minute)^6^. Connected speech intelligibility was fair-good. In summary, the patient presented with moderate mixed spastic-flaccid dysarthria.

References

1. Roussel, P. *et al.* Observation and assessment of acoustic contamination of electrophysiological brain signals during speech production and sound perception. *J. Neural Eng.* **17**, 056028 (2020).

2. Lapuschkin, S. *et al.* Unmasking Clever Hans predictors and assessing what machines really learn. *Nat. Commun.* **10**, 1096 (2019).

3. Cedarbaum, J. M. *et al.* The ALSFRS-R: a revised ALS functional rating scale that incorporates assessments of respiratory function. *J. Neurol. Sci.* **169**, 13–21 (1999).

4. Kent, R. D., Kent, J. F. & Rosenbek, J. C. Maximum Performance Tests of Speech Production. *J. Speech Hear. Disord.* **52**, 367–387 (1987).

5. Pierce, J. E., Cotton, S. & Perry, A. Alternating and sequential motion rates in older adults. *Int. J. Lang. Commun. Disord.* **48**, 257–264 (2013).

6. Darley, F. L. & Spriesterbach, D. C. *Diagnostic Methods in Speech Pathology*. (Harper & Row, New York, NY, 1978).
