# Supplementary material for: Online speech synthesis using a chronically implanted brain–computer interface in an individual with ALS
Source: Sci Rep. 2024 Apr 26;14:9617. doi: 10.1038/s41598-024-60277-2 (PMC11053081; doi:10.1038/s41598-024-60277-2)
Supplement: Supplementary file 3 — Supplementary Legends. [file 41598_2024_60277_MOESM3_ESM.docx]

## Supplementary Video

This study is accompanied by a video of the participant during one block of a closed-loop session, which demonstrates identification of speech segments from the participant through the unidirectional voice activity detection RNN and closed-loop reconstruction of the spoken speech signal. Note that we masked out the delayed feedback from the patient’s speech, which was recorded from the microphone while played back on the loudspeaker. Instead, we incorporated the same acoustic signal that was played back on the loudspeaker as a separate channel described as BCI (dark red).
